# The TGF-β1/p53/PAI-1 Signaling Axis in Vascular Senescence: Role of Caveolin-1

**DOI:** 10.3390/biom9080341

**Published:** 2019-08-03

**Authors:** Rohan Samarakoon, Stephen P. Higgins, Craig E. Higgins, Paul J. Higgins

**Affiliations:** Department of Regenerative & Cancer Cell Biology, Albany Medical College, Albany, NY 12208, USA

**Keywords:** vascular disease, senescence, p53, TGF-β1, plasminogen activator inhibitor-1

## Abstract

Stress-induced premature cellular senescence is a significant factor in the onset of age-dependent disease in the cardiovascular system. Plasminogen activator inhibitor-1 (PAI-1), a major TGF-β1/p53 target gene and negative regulator of the plasmin-based pericellular proteolytic cascade, is elevated in arterial plaques, vessel fibrosis, arteriosclerosis, and thrombosis, correlating with increased tissue TGF-β1 levels. Additionally, PAI-1 is necessary and sufficient for the induction of p53-dependent replicative senescence. The mechanism of PAI-1 transcription in senescent cells appears to be dependent on caveolin-1 signaling. *Src* kinases are upstream effectors of both FAK and caveolin-1 activation as FAK^Y577,Y861^ and caveolin-1^Y14^ phosphorylation are not detected in TGF-β1-stimulated *src* family kinase (pp60^c-*src*^, Yes, Fyn) triple-deficient (SYF^−/−/−^) cells. However, restoration of pp60^c-src^ expression in SYF-null cells rescued both caveolin-1^Y14^ phosphorylation and PAI-1 induction in response to TGF-β1. Furthermore, TGF-β1-initiated *Src* phosphorylation of caveolin-1^Y14^ is critical in Rho-ROCK-mediated suppression of the SMAD phosphatase PPM1A maintaining and, accordingly, SMAD2/3-dependent transcription of the PAI-1 gene. Importantly, TGF-β1 failed to induce PAI-1 expression in caveolin-1-null cells, correlating with reductions in both Rho-GTP loading and SMAD2/3 phosphorylation. These findings implicate caveolin-1 in expression controls on specific TGF-β1/p53 responsive growth arrest genes. Indeed, up-regulation of caveolin-1 appears to stall cells in G_0_/G_1_ via activation of the p53/p21 cell cycle arrest pathway and restoration of caveolin-1 in caveolin-1-deficient cells rescues TGF-β1 inducibility of the PAI-1 gene. Although the mechanism is unclear, caveolin-1 inhibits p53/MDM2 complex formation resulting in p53 stabilization, induction of p53-target cell cycle arrest genes (including PAI-1), and entrance into premature senescence while stimulating the ATM→p53→p21 pathway. Identification of molecular events underlying senescence-associated PAI-1 expression in response to TGF-β1/*src* kinase/p53 signaling may provide novel targets for the therapy of cardiovascular disease.

## 1. Introduction: Vascular smooth muscle cell (VSMC) Senescence and Vascular Disease

Tissue-resident cells acquire the morphologic and biochemical hallmarks of a senescent phenotype during development of several age-related disorders including atherosclerosis, idiopathic lung fibrosis, chronic obstructive pulmonary disease, osteoarthritis, Alzheimer’s and Parkinson’s disease, cancer, and renal fibrosis [1,2,3,4]. In the cardiovascular system, stress-induced premature cellular senescence is a major contributor to the onset of age-dependent pathologies and compromised vessel function [4,5]. Vascular disease remains the predominant cause of mortality worldwide, particularly in western industrialized countries where chronological age is the most significant risk determinant [6]. Estimates suggest that more than one-third of the population 40 to 60 years old and 83% of those over 85 have some form of cardiovascular disease [4].

Stable cell cycle arrest, induced by intrinsic or extrinsic stress, defines the emergence of senescent cells during organismal aging. Typical senescence-inducing stimuli include telomere erosion due to repeated cell division (replicative senescence), generation of reactive oxygen species (ROS), DNA damage (genotoxic stress) and the activation of certain transforming genes (oncogene-induced senescence) [7,8,9]. Although expression of β-galactosidase is a commonly used indicator, telomere erosion, chromatin fragmentation and presence of heterochromatin foci provide more mechanistically linked, DNA-level molecular biomarkers of the senescent phenotype [10,11,12]. 

**Figure 1:**
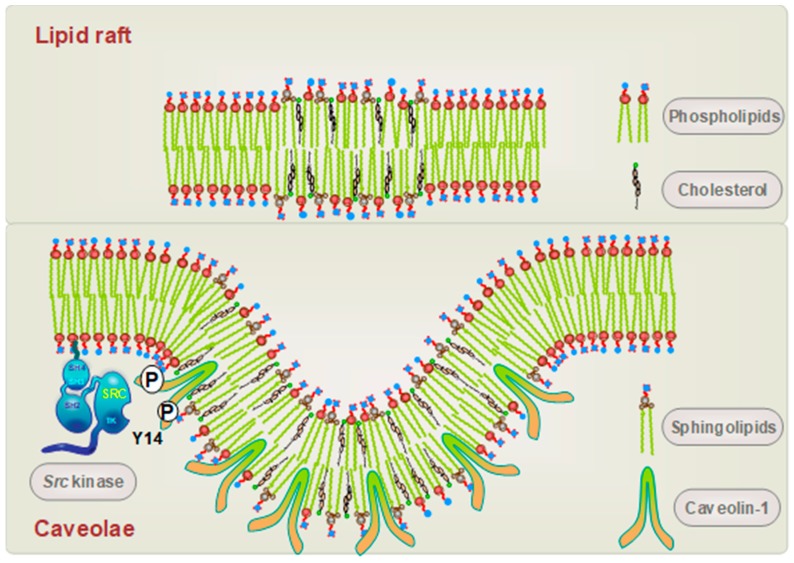
Comparative structures of lipid rafts and caveolae, two major cholesterol-rich membrane microdomains. Caveolin-1 is a key membrane protein necessary for the formation of cholesterol- and sphingolipid-enriched caveolae. *Src* family kinases phosphorylate caveolin-1 at Y14 promoting interactions with a subgroup of signaling effectors including the EGFR and FAK, as seen in Figure 2.

Vascular senescence and subsequent lineage-specific cellular dysfunction is a fundamental aspect of several age-related cardiovascular disorders [13]. Progressive arterial non-compliance, due to increased tunica media collagen synthesis by aging vascular smooth muscle cells (VSMCs), results in the development of hypertension and associated pathologic sequalae. Accelerated VSMC aging in atherosclerosis results from replicative stress (i.e., reduced telomere integrity) or premature senescence due to elevated levels of ROS and DNA damage [5,14]. Plaque-derived VSMCs display typical senescence-like morphologic features (e.g., large, flattened cell shape) and have a restricted ability to proliferate in vitro (e.g., G1 stalling or arrest, reduced S phase cohort) and shortened life span [15]. The process of biological aging appears to be increased in various cell types that comprise the plaque, with VSMCs acquiring disease biomarkers and negative growth cycle regulators, particularly in advanced lesions, that contribute to disease progression, plaque rupture, and adverse clinical outcomes [13,16,17]. Caveolin-1, the major structural protein of cholesterol-rich plasma membrane invaginations or caveolae, as seen in Figure 1, is one such member of the network of factors that positively impacts stress-induced premature senescence in age-associated disease and is viewed as a “gatekeeper” of cellular senescence as well as an aging biomarker [18]. Although the mechanism is not fully understood, caveolin-1 appears involved in the expression of a subgroup of the senescence-associated secretory phenotype (SASP) gene set [19,20], several of which control the inflammatory and proliferative response to TGF-β1, a significant contributor to vascular sclerosis [21]. 

Plaque-associated VSMCs exhibit other molecular traits characteristic of the senescent state including induction of the cell cycle inhibitors p16 and p21, reduced E2F transcriptional activation of proliferation-promoting genes due to retinoblastoma (Rb) protein hypophosphorylation and up-regulation of p53 [14,15,16,22]. There is some controversy as to the actual role of p53 in age-related disorders [13]; however, p53 is a critical factor in vessel pathophysiology and down-modulates cellular levels of the telomere protective repeat-binding protein 2 (TRF2), likely via a p53-dependent ubiquitin ligase [23]. Plaque VSMCs have shortened telomeres and decreased TRF2 levels. Overexpression of a functional TRF2 construct results in a senescence bypass, reduces DNA damage, accelerates repair, and attenuates the extent of vascular disease compared to a loss-of-function TRF2 mutant which exacerbates atherosclerosis and necrotic core formation [23]. p53 activation also sensitizes VSMCs to Fas-mediated apoptosis by increasing cell surface Fas levels. This has significant consequences; unlike viable VSMCs which promote plaque stability via maintenance of collagen expression, a major determinant of the fibrous cap tensile strength perhaps in response to elevations in vessel TGF-β levels, apoptotic VSMCs contribute to plaque vulnerability while simultaneously functioning as nucleating structures for vascular calcification [14]. Indeed, senescent VSMCs overexpress osteoblastic genes (e.g., alkaline phosphatase, RUNX-2, type I collagen, BMP-2) suggesting that acquisition of a pro-calcificatory phenotype is one aspect of a cell type-exclusive senescence program in the vascular system [24,25].

## 2. Caveolin-1 and the Senescent Phenotype: Involvement of p53

Up-regulation of caveolin-1 is evident in senescent cells and linked to several age-related diseases [18,26]. Overexpression approaches implicate caveolin-1 in the development of premature senescence in primary fibroblasts [27], the acquisition of senescence-associated morphologic restructuring [28], and the stalling of cells in G_0_/G_1_ by activation of the p53/p21 cell cycle arrest pathway [29]. The precise mechanism(s) underlying caveolin-1-associated p53 mobilization and involvement in cellular senescence may vary as a function of cell type and the nature of the arrest state, oxidative stress promotes interactions between caveolin-1 and the dual-site E3 ubiquitin ligase mouse double minute 2 homolog (MDM2); however, a major negative regulator of p53 transcriptional activity and stability [30]. The MDM2 caveolin-1 binding domain overlaps with the p53 binding motif of MDM2, suggesting that caveolin-1 may inhibit p53/MDM2 complex formation resulting in p53 stabilization, upregulation of p53-target growth arrest genes (e.g., p21, plasminogen activator inhibitor-1), and entrance into premature senescence [26,31,32]. Introduction of a peptide corresponding to the MDM2 binding region of caveolin-1 has a comparable effect while oxidative stress–induced activation of the p53/p21pathway and senescence induction are mitigated in caveolin-1-null mouse embryonic fibroblasts [32]. Enhanced interaction between caveolin-1 and MDM2 is also evident in diabetic fibroblasts which have a significantly decreased antioxidant capacity and exhibit both a heightened level of oxidative stress and a senescence-like phenotype [33]. Caveolin-1 knockdown attenuates diabetes-associated premature senescence and p53/MDM2 complex formation. However, ATM is also a critical regulator of stress-induced p53 and the p53 signaling pathway [2]. Caveolin-1 activates ATM likely by sequestering PP2A, which negatively regulates ATM autophosphorylation, into caveolar structures thereby stimulating engagement of the ATM→p53→p21 pathway [26]. These data indicate that, under conditions of oxidative stress-induced senescence, caveolin-1 down-regulates p53/MDM2 binding while promoting ATM function collectively activating p53/p21 signaling and subsequent growth arrest. Although specific clinical-grade drugs are not yet available, senolytic targeting of the p53/p21 network, perhaps via inhibition of p53/FOXO4 interactions or knockdown of the p53 responsive p21 gene [34], may have potential clinical utility in the context of caveolin-1-induced vascular cell senescence and vessel pathology. The therapeutic implications of such approaches may also be relevant to p53-associated mechanisms of vascular disease other than those initiated as a consequence of caveolin-1 deregulation. Indeed, expression of the actin-binding smooth muscle protein SM22α in VSMCs is increased upon chronic Angiotensin II treatment [35]. SM22α overexpression induces VSMC senescence, a response that is p53 dependent. Elevated levels of SM22α inhibit p53 ubiquitination and degradation by attenuating PI3K/Akt-mediated phosphorylation and activation of MDM2 decreasing interactions between MDM2 and p53 resulting in p53 stabilization [35]. 

## 3. Caveolin-1 Signaling Is Required for Expression of the Senescence-Inducing p53-Target PAI-1 Gene in VSMCs

Plasminogen activator inhibitor type-1 (PAI-1, SERPINE1) is a major promoter of vascular-related pathologies, including arterial thrombosis and perivascular fibrosis [36,37] as well as a biomarker and prognostic indicator of TGF-β1-stimulated neointima formation and disease progression [21,36,38,39,40]. In some cell types, including VSMCs, TGF-β1 functions as a senescence driver. Recent findings suggest that TGF-β1 induces VSMC senescence through reactive oxygen species-stimulated activation of the NF-κB signaling pathway and expression of SASP factors, including PAI-1 [41,42]. Transgenic mice that overexpress PAI-1 develop age-dependent atherosclerosis while PAI-1-deficient animals are protected from experimentally induced vascular disease [37]. Importantly, PAI-1 is not merely a biomarker of the senescent phenotype but is necessary and sufficient for the induction of replicative senescence downstream of p53 [43] and is a key inducer of the senescence program [44]. Indeed, PAI-1 is consistently among the highest up-regulated genes in all models of induced senescence, confirming its causative involvement in age-related disease [45,46]. Consistent with this implication, a loss of function frameshift mutation in the PAI-1 gene in an Old Order Amish community is associated with telomere length, lower incidence of diabetes, and longer life span [47]. Moreover, exogenously delivered PAI-1 alone stimulates TGF-β1 synthesis, suggesting the existence of a PAI-1/TGF-β1-positive feed-forward mechanism [48]. This provides for a model whereby elevated tissue levels of TGF-β1 during the emergence of the senescent phenotype stimulate expression of PAI-1 that, in turn, reinforces continued TGF-β1 synthesis promoting the maintenance, and perhaps expansion, of the senescent VSMC population. Targeted down-modulation of PAI-1 expression or function may be one approach to inhibit the onset and progression of vascular fibrosis. 

**Figure 2:**
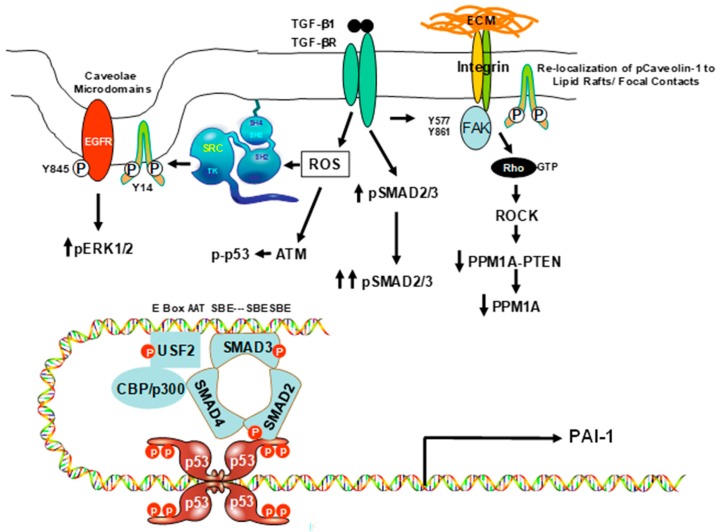
A model illustrating the maintenance of induced SMAD3 phosphorylation and PAI-1 transcription in response to TGF-β1 via *Src*/p53/FAK/Caveolin-1 signaling. Binding of TGF-β1 to the TGF-β receptor (TGF-βR) stimulates the generation of reactive oxygen species (ROS) and activates *Src* kinases. *Src* phosphorylates caveolin-1 at Y14 and transactivates the EGFR at the *Src* target Y845 residue, leading to mobilization of the MEK-ERK and p38 (not shown) pathways. TGF-β1-initiated *Src* kinase phosphorylation of caveolin-1^Y14^ also stimulates FAK activation, Rho-GTP loading, and Rho-ROCK activation at sites of integrin/matrix engagement. pCaveolin-1^Y14^-Rho-ROCK signaling inhibits PTEN-PPM1A interactions, resulting in a reduction of the SMAD phosphatase PPM1A, maintaining pSMAD2/3 levels required for PAI-1 induction and persistent expression in response to TGF-β1. ROS-mediated ATM activation stimulates p53 phosphorylation and recruitment of p-p53 to the promoter region of genes with p53 binding motifs [31,51,53,59]. The PE2 region E box in the PAI-1 promoter is a docking site for the helix-loop-helix transcription-leucine zipper factors USF1/2 which are activated by MAP kinases as well as other TGF-β1-induced kinases. Members of the USF family reorient the DNA minor grove, promoting interactions between SMAD2/3 tethered to the PE2 region SMAD-binding elements (SBEs) with tetramerized p53, bound to its downstream half-site motifs [51,59]. Occupancy of the immediate 5” upstream SMAD-binding elements (SBEs) with SMAD2/3/4 and co-localization with p53, USF2, and the histone acetyltransferases CBP/p300 facilitates the formation of a multi-component transcriptional complex required for TGF-β1-induced PAI-1 expression.

The mechanism of PAI-1 transcriptional activation in cellular senescence [49,50] is less clear but appears to be dependent on caveolin-1 signaling [51], as seen in Figure 2. Cooperation between non-SMAD (i.e., pp60^c-src^- EGFR-ERK1/2) pathways and SMAD signaling is necessary for maximal TGF-β1-stimulated PAI-1 transcription [52,53,54,55]. TGF-β1-induced Rho-ROCK activation, for example, affects the duration, but not the initiation, of SMAD2/3 phosphorylation; however, the underlying molecular basis and impact on TGF-β1 target gene expression is unknown [51]. Furthermore, TGF-β1-mediated Rho-activation is repressed in caveolin-1-null cells, perhaps due to deficient caveolin-1/caveolae-dependent TGF-β1 receptor interactions and internalization [56]. However, Caveolin-1 appears necessary for TGF-β1-mediated fibronectin expression in mesangial cells, suggesting that caveolin-1 regulation of TGF-β1 signaling may be context-dependent as well as cell type-specific. This possibility is supported by an analysis of conflicting findings. In some cells, caveolin-1 appears to attenuate TGF-β signaling through interaction with the TGF-β type I receptor inhibiting SMAD2/3 phosphorylation, down-regulation of TGF-β type II receptor expression, increased receptor internalization as well as degradation and interference with latent TGF-β activation [57]. In mouse embryonic fibroblasts, caveolin deficiency results in acquisition of a senescent-like phenotype through the p53-p21 pathway [58]; however, in primary fibroblasts, overexpression of caveolin-1 induces the p53-p21 growth arrest program [27,28,29]. TGF-β1 activates p53 and both caveolin-1 and p53 are required for expression of the profibrotic p53 target PAI-1 gene and VSMC senescence [51,59]; accordingly, there are considerable cell type-dependent controls on the specific involvement of caveolin-1 in growth regulation, entry into a senescent state, and transcription of cell cycle arrest genes.

## 4. Mechanisms of Caveolin-1 Signaling

Caveolin-1 scaffolds spatially position and functionally regulate an increasing repertoire of effector molecules [60,61]; however, the available data differ regarding mechanism and imply that the specific role of caveolin-1 in signaling may be cell lineage-dependent. SMAD2/3 phosphorylation and PAI-1 induction in response to TGF-β1 is suppressed by genetic deficiency of caveolin-1 [51,62], likely a result of elevated expression of the transcriptional co-repressor SnoN [62], implicating caveolin-1 as an activator of the Rho-ROCK-SMAD2/3 pathway [51]. Significantly reduced SMAD3 phosphorylation and increased expression of the SMAD phosphatase PPM1A expression in triple-deficient (pp60^c-*src*^, Yes, Fyn) *src* kinase (SYF^−/−/−^) cells correlates with reduced PAI-1 levels [51]. *Src* kinase-mediated FAK^Y577 and Y861^ phosphorylation is stimulated by TGF-β1; although FAK^Y397^ autophosphorylation is *src*-independent, FAK is both involved in and required for caveolin-1^Y14^ phosphorylation, pSMAD3 activation, and PAI-1 expression [51]. Genetic reprogramming in response to TGF-β1utilizes SMAD (canonical) as well as non-SMAD (non-canonical) pathways [19,53,63,64,65]; although the role of SMADs as transcriptional effectors of TGF-β1signaling is well established, how non-SMAD elements (e.g., Rho-ROCK, *src* kinases, FAK, caveolin-1) integrate into the more traditional canonical SMAD pathways is likely cell type- and target gene-dependent. *Src* kinases are critical upstream regulators of FAK and caveolin-1 activation as FAK^Y577^ and FAK^Y861^ and caveolin-1^Y14^ phosphorylation are undetectable in TGF-β1 stimulated SYF^−/−/−^ mouse embryo fibroblasts. However, pp60*^c-src^* restoration in the SYF-deficient genetic background rescues both TGF-β1-induced caveolin-1^Y14^ phosphorylation and PAI-1 expression [51]. Although the PAI-1 gene is unresponsive to TGF-β1 stimulation in caveolin-1-null fibroblasts, re-expression of a caveolin-1 construct in caveolin-1^-/-^ cells rescues PAI-1 inducibility. The mechanism of gene regulation downstream of caveolin-1 is unclear; however, TGF-β1 promotes interaction between RhoA and caveolin-1 leading to RhoA activation. Such synergy is attenuated in caveolin-1-deficient fibroblasts [51,57]. Moreover, TGF-β1-dependent PAI-1 expression in hepatic epithelial cells similarly requires cooperation between caveolin-1 and SMAD2/3 [62]. Finally, TGF-β1-initiated mobilization of the *src*-FAK-caveolin-1-SMAD3 pathway and target gene transcription requires generation of reactive oxygen species (ROS) linking modulation of the cellular redox state to gene reprogramming [51], similar to events underlying development of the senescent VSMC phenotype [41,42]. 

## 5. Controversies and Conclusions

Spatiotemporal signal switching, e.g., [66] involving the redirection, duration, and compartmentalization of caveolin-1 participation in EGFR versus RhoA activation and EGFR, *src* kinase p53 mobilization, likely dictates the specific caveolin-1 contribution to the senescent versus proliferative phenotypes. Outcome confounders include the cell type, whether normal or transformed, degree of “plasticity”, the networks engaged, and the level of caveolin-1 expressed e.g., [67]. Indeed, caveolin-1 deficiency can also induce cellular senescence by initiating mitochondrial dysfunction, impairing mitochondrial respiration and inactivating SIRT1 [68]. This pathway, like the p53-dependent transcriptional response [43], also involves activation of the p53→p21 growth arrest program [58]. 

**Figure 3:**
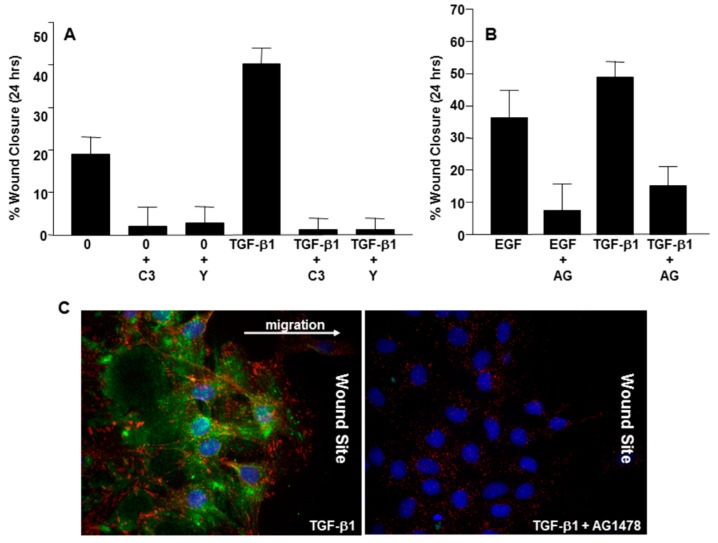
Signaling requirements for VSMC monolayer scratch would closure. Confluent VSMC cultures were scrape-wounded with a pipette tip prior to addition TGF-β1 or EGF with (+) or without a 30 min pre-incubation with the Rho GTPase inhibitor C3 transferase (C3), the ROCK inhibitor Y-27632, or the EGFR kinase inhibitor AG1478. C3 transferase and Y-27632 effectively attenuated both basal and TGF-β1-stimulated VSMC migration (**A**) and AG1478 significantly reduced EGF as well as TGF-β1 induced migration (**B**) in response to monolayer wounding. In TGF-β1-treated cultures, PAI-1 expression was evident at the wound edge within hours post-injury (**C**, **left panel**). PAI-1 induction was completely inhibited by exposure to AG1478 (**C**, **right panel**) correlating with a significant reduction in cell migration (**B**).

The caveolin-1/*src* kinase/EGFR triad involves interaction of the EGFR with caveolin-1 and phosphorylation of caveolin-1^Y14^ by *src* [68,69,70] and can both positively and negatively regulate EGFR activation e.g., [51,70]. The mechanisms underlying these divergent findings are unclear but likely are a result of the differences in cell type and the pathways impacted. However, Caveolin-1 is a transcriptional target of hypoxia-inducible factors 1 and 2, resulting in increased EGFR dimerization within caveolae and ligand-independent receptor activation stimulating cell proliferation and migration [71]. EGF signaling to RhoA also requires *src* kinases, is galectin 3-/p-caveolin-1^Y14^-dependent, and leads to increased cell migration [72]. This pathway induces actin reorganization, RhoA activation, and acquisition of a motile phenotype [72]. In this model, EGF stimulates *src*-mediated caveolin-1 phosphorylation, perhaps as a consequence of *src* activation by TGF-β1 (53), and RhoA/ROCK signaling that, in turn, promotes EGF- as well as TGF-β1-induced VSMC migration, as seen in Figure 3. However, other mechanisms of pathway mobilization are also evident. The apoptosis-inducing ligand TRAIL promotes the localization and activation of *src* kinase into lipid raft structures, facilitating complex formation with both the EGFR and caveolin-1 [73]. A *src* inhibitor blocked such interactions while caveolin-1 knockdown attenuated EGFR phosphorylation. Moreover, desmoglein 2 recruits caveolin-1, EGFR and *src* to membrane rafts and modulates EGFR signaling; overexpression of desmoglein 2 enhances EGFR activation and increases *src*-/EGFR-dependent cell proliferation and migration [74]. Similarly, TGF-β1 induces PAI-1 expression and the TGF-β1- or EGF-mediated acquisition of a motile phenotype in VSMCs involves pp60^c-*src*^, EGFR^Y845^, and RhoA/ROCK signaling; at the same time, caveolin-1 is required for TGF-β1-initiated EGFR transactivation [51,53,75]. Even publications that report divergent findings related to the involvement of caveolin-1 in EGF-dependent EGFR activation and cell migration, a key aspect of VSMC dysfunction in cardiovascular disease, conclude that caveolin-1 overexpression inhibits growth of various cells including VSMCs [76,77,78], a prelude to entry into a senescent end-state.

Vascular cell senescence is a common finding in several aging-related cardiovascular disorders [13]; accordingly, one therapeutic approach involves administration of senolytic drugs, either individually or in combination, that specifically target senescent cells. Although initial findings were disappointing, recent studies suggest that eliminating senescent cells improves organ function and prolongs lifespan [79,80]. Small molecule delivery successfully eliminated p16^Ink4a^ overexpressing senescent cells [81]. Suicide gene therapy, directed against proteins that comprise the senescence signature, appears more efficacious in clearing p16^Ink4a^-overexpressing tumor cells (with mutations in downstream effectors of p16^Ink4a+^ such as RB and CDK4/6) as well as normal senescent cells than direct attempts to eliminate these cells with senolytic drugs [82]. Whether similar strategies can be adapted in the context of the caveolin-1-associated vascular senescence remains to be determined. Collectively, these findings suggest that continued identification of molecular mechanisms underlying caveolin-1-dependent senescence-associated signaling pathways (e.g., TGF-β1/*src* kinase/p53) that regulate expression of specific caveolin-1-regulated senescence-conferring genes (e.g., PAI-1) may provide novel targets for the therapy of cardiovascular disease.
